# Affective Valence and Enjoyment in High- and Moderate-High Intensity Interval Exercise. The Tromsø Exercise Enjoyment Study

**DOI:** 10.3389/fpsyg.2022.825738

**Published:** 2022-03-22

**Authors:** Tord Markussen Hammer, Sigurd Pedersen, Svein Arne Pettersen, Kamilla Rognmo, Edvard H. Sagelv

**Affiliations:** ^1^School of Sport Sciences, Faculty of Health Sciences, UiT The Arctic University of Norway, Tromsø, Norway; ^2^Department of Psychology, Faculty of Health Sciences, UiT The Arctic University of Norway, Tromsø, Norway

**Keywords:** training, adherence, public health, emotion, PACES

## Abstract

**Introduction:**

Exercise at high intensity may cause lower affective responses toward exercise compared with moderate intensity exercise. We aimed to elucidate affective valence and enjoyment in high- and moderate-high interval exercise.

**Methods:**

Twenty recreationally active participants (9 females, 11 males, age range: 20–51 years) underwent three different treadmill running exercise sessions per week over a 3-week period, in randomized order; (1) CE70: 45 min continuous exercise at 70% of heart rate maximum (HR_max_), (2) INT80: 4 × 4 min intervals at 80% of HR_max_, (3) INT90: 4 × 4 min intervals at 90% of HR_max_. Pre-tests included graded submaximal steady state intensities and a test to exhaustion for determining peak oxygen uptake and HR_max_. Affective valence (pleasure/displeasure) was measured before, during and after the sessions using the Feeling Scale (FS). Enjoyment was assessed before and after the sessions applying the Physical Activity Enjoyment Scale (PACES) and during the sessions using the Exercise Enjoyment Scale (EES).

**Results:**

The participants felt lower pleasure (between-sessions effect: *p* = 0.02, _*p*_η^2^: 0.13) during INT90 sessions (FS: 1.08, 95% CI: 0.35–1.92) compared with INT80 (FS: 2.35, 95% CI: 1.62–3.08, *p* = 0.052) and CE70 sessions (FS: 2.45, 95% CI: 1.72–3.18, *p* = 0.03), with no differences between INT80 and CE70 sessions (*p* = 1.00). There were higher enjoyment after INT80 sessions (PACES: 101.5, 95% CI: 95.7–107.3) versus CE70 sessions (PACES: 91.3 95% CI: 85.5–97.1, *p* = 0.046), and no differences between INT90 (PACES: 98.2, 95% CI: 92.4–103.4) and CE70 (*p* = 0.29) or INT80 (*p* = 1.00). For enjoyment during exercise, CE70 were perceived more enjoyable, and INT80 and INT90 less enjoyable in week 2 (EES: week x session: *p* = 0.01, _*p*_η^2^: 0.11; CE70: 4.3, 95% CI: 3.6–4.9, INT80: 4.6, 95% CI: 3.9–5.2, INT90: 4.0, 95% CI: 3.4–4.7) and 3 (EES: CE70: 4.2, 95% CI: 3.7–4.8, INT80: 4.8, 95% CI: 4.2–5.3, INT90: 4.3, 95% CI: 3.8–4.9) than in week 1 (EES: CE70: 3.5, 95% CI: 3.0–4.0, INT80: 5.0, 95% CI: 4.5–5.5, INT90: 4.5, 95% CI: 4.0–5.0).

**Conclusion:**

The negative affective consequences associated with high intensity interval exercise can be alleviated by keeping the intensity at or around 80% of HR_max_ while preserving the beneficial enjoyment responses associated with interval exercise.

## Introduction

Adherence to exercise programs is low and influenced by multiple personal and demographic factors (Stutts, 2002; Trost et al., 2002; Sequeira et al., 2011; Picorelli et al., 2014). Common reported obstacles for individuals are lack of time for- and enjoyment of exercise (Stutts, 2002; Sequeira et al., 2011). This has brought forth an interest among researchers to design effective exercise programs that are both enjoyable and time efficient (Oliveira et al., 2018).

Exercise enjoyment during and after exercise could be a mediating factor for exercise adherence (Raedeke, 2007; Ekkekakis, 2009; Jekauc, 2015), as greater enjoyment increases the likelihood of performing regular exercise (Salmon et al., 2003; Lewis et al., 2016). Exercise enjoyment after exercise, measured with the physical activity enjoyment scale (PACES) questionnaire (Kendzierski and DeCarlo, 1991), is generally reported to be higher following high intensity interval exercise compared with moderate intensity continuous exercise (Oliveira et al., 2018; Niven et al., 2020; Tavares et al., 2021). Furthermore, the enjoyment of high intensity interval exercise may improve with familiarity to the exercise modality (Smith-Ryan, 2017). A previous six weeks long study observed an initial similar exercise enjoyment between high intensity intervals [95–95% of peak heart rate (HR_*peak*_)] and moderate intensity continuous exercise (70–75% of HR_*peak*_), but the enjoyment progressively increased over 6 weeks for those performing interval exercise, resulting in higher enjoyment compared with continuous exercise following 4 weeks of the intervention (Heisz et al., 2016). However, such time effects in enjoyment are not consistent across studies (Kong et al., 2016; Vella et al., 2017).

While enjoyment is a specific feeling evaluated by cognition, affective responses are reflexive responses for the direction of emotion (pleasure/displeasure) (Ekkekakis, 2013), and may also mediate exercise adherence (Rhodes and Kates, 2015). The two concepts are distinctively separate, but also closely related, where higher exercise enjoyment can promote positive affect and *vice versa* (Raedeke, 2007; Jekauc, 2015). The affective responses to exercise seem influenced by exercise intensity, where higher intensities increase displeasure (i.e., negative affect) (Ekkekakis and Petruzzello, 1999; Welch et al., 2007), at least when exceeding physiological markers of increased relative contribution of anaerobic metabolism (Ekkekakis et al., 2011).

The transition toward a higher relative contribution from anaerobic energy systems in terms of an anaerobic threshold (AT) have provided controversy and confusion (Hopker et al., 2011; Poole et al., 2021). Indeed, different thresholds intended to pinpoint processes associated with a relatively higher contribution from anaerobic metabolism (e.g., ventilatory threshold, lactate threshold, onset of blood lactate accumulation (OBLA), maximal lactate steady state) correspond to a range of different relative intensities in terms of percentage of maximal oxygen uptake (VO_2max_) and maximal heart rate (HR_max_) (Faude et al., 2009; Poole et al., 2021). The maximal lactate steady state running speed may be the most precise estimate of an AT but require multiple exercise testing sessions (Jamnick et al., 2018), making it less feasible compared with OBLA-defined estimates determined by a single graded exercise test. Of the suggested AT estimates available in the literature, an OBLA at 2.5 mmol/L blood lactate concentration (BLa) was identified as an acceptable AT definition compared with the maximal lactate steady state (Jamnick et al., 2018).

Recent meta-analyses indicate that high intensity interval exercise generates more negative affective responses than moderate intensity continuous exercise, while at the same time evoking higher enjoyment (Niven et al., 2020; Tavares et al., 2021). The intermittent nature of interval exercise provides a constantly changing stimulus, making it less monotonous, and potentially a more enjoyable experience than continuous exercise (Thum et al., 2017). Consequently, it may constitute that performing interval exercise with lower contribution from anaerobic metabolism, compared with higher, generates higher positive affect. At the same time, the intermittent structure of interval exercise may provide a more enjoyable experience than continuous exercise. Previous studies have compared high intensity interval exercise with moderate intensity continuous exercise, while to our knowledge fewer studies have compared interval exercise at different intensities (Boyd et al., 2013; Olney et al., 2018).

Moreover, lower enjoyment is reported if the interval exercise intensity is too strenuous to complete (Oliveira et al., 2013; Martinez et al., 2015). Here, higher volume high intensity intervals with long interval bouts, such as 4 × 4 min intervals (total time at high intensity: 16 min) at ∼90% of HR_max_, are designed to not be totally exhaustive and thus could be a viable option for exercise adherence (Taylor et al., 2019). Such long interval bouts are not supramaximal, and demand lower anaerobic metabolism compared with shorter interval (<2 min) bouts with supramaximal intensities at >100% of VO_2max_ (Wiewelhove et al., 2016; Wood et al., 2016; Valstad et al., 2018). To the best of our knowledge, no previous studies have assessed affective valence and enjoyment responses to long interval bouts at different intensities.

Thus, the aims of this study were (1) to compare affective valence and enjoyment responses before, during and after treadmill running exercise when performed as 4 × 4 min intervals at moderate-high intensity (80% of HR_max_) and high intensity (90% of HR_max_) and as 45 min moderate intensity continuous exercise (70% of HR_max_), with each exercise modality performed once a week over three weeks, and (2) to assess the associations between percentage of individual AT running speed and percentage of individual AT Rating of Perceived Exertion (RPE) (corresponding to OBLA at 2.5 mmol/L) and affective valance and enjoyment during and after treadmill running exercise.

## Materials and Methods

### Design

**FIGURE 1:**
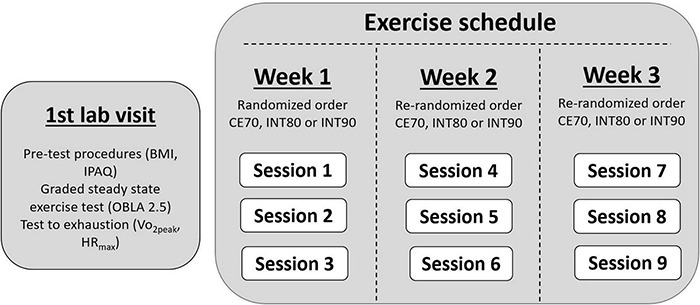
Baseline testing and three-week exercise schedule. BMI, Body Mass Index; IPAQ, International Physical Activity Questionnaire; OBLA 2.5, Onset of blood lactate accumulation at 2.5 mmol/L; VO_2peak_, Peak Oxygen Uptake; HR_max_, maximal heart rate; CE70, moderate intensity continuous exercise at 70% of HR_max_; INT80, 4 × 4 min intervals at 80% of HR_max_; INT90, 4 × 4 min intervals at 90% of HR_max_.

In this randomized crossover study, 20 participants performed three different treadmill running exercise sessions per week over three weeks (3 sessions × 3 weeks = 9 sessions in total), in randomized order for each week; (1) CE70: 45 min moderate intensity continuous exercise at 70% of HR_max_, (2) INT80: 4 × 4 min moderate-high intensity interval exercise at 80% of HR_max_ interspaced with 3 min moderate intensity exercise at 70% of HR_max_ between bouts and (3) INT90: 4 × 4 min high intensity interval exercise at 90% of HR_max_ interspaced with 3 min moderate intensity exercise at 70% of HR_max_ between bouts. Prior to the exercise sessions, the participants performed a graded steady state treadmill running test to determine the AT, defined as the running speed and RPE associated with 2.5 mmol blood lactate concentration (BLa) in whole blood, followed by a test to exhaustion to determine peak oxygen uptake (VO_2*peak*_) and HR_max_. The schedule for baseline testing and exercise sessions are presented in Figure 1.

Immediately prior to each exercise session, the participants rated their affective valence and perceived enjoyment toward the upcoming session by answering the Feeling Scale (FS) (Rejeski et al., 1987; Hardy and Rejeski, 1989) and the PACES (Kendzierski and DeCarlo, 1991), respectively. After 55% completion of each exercise session (while still exercising), the participants answered the FS again, rated their perceived enjoyment using the Exercise Enjoyment Scale (EES) (Stanley and Cumming, 2010), and rated their perceived exertion using Borg’s RPE 6–20 scale (Borg, 1974). Finally, immediately following completion of each exercise session, the participants answered the FS, PACES and rated their RPE.

### Participants

Twenty-four recreationally active participants (9 females, 11 males, age range: 20-51 years) were recruited through stands and posters at the Tromsø campus of UiT the Arctic University of Norway and social media campaigns. Four of the participants withdrew from the study (reported reasons: lack of time and injuries not related to the study’s intervention). Three participants underwent eight out of nine exercise sessions, where their final exercise session (two INT90 and one CE70) was canceled due to COVID-19 lockdown of university facilities at 12th of March 2020. For these three individuals, we performed intention to treat analyses by forwarding their respective mean score (CE70 or INT90) of week 1 and 2 to their respective missing exercise session in week 3. Consequently, we ended up with a sample of 20 participants for our final analyses. The participants’ characteristics are presented in Table 1.

**TABLE 1:** Descriptive characteristics.

	Women (*n* = 9)	Men (*n* = 11)	Total (*N* = 20)
Age (years) Minimum-maximum	31.2 ± 13.7 20–51	22.8 ± 3.3 20–32	26.6 ± 10.2 20–51
Weight (kg) Minimum-maximum	63.7 ± 6.2 55.7–75.0	80.3 ± 9.8 68.3–100.5	72.87 ± 11.75 55.7–100.5
Height (m) Minimum-maximum	1.68 ± 0.04 1.63–1.74	1.79 ± 0.06 1.68–1.86	1.74 ± 0.07 1.63–1.86
BMI (kg/m^2^) Minimum-maximum	22.6 ± 2.4 20.1–27.9	25.3 ± 3.5 21.0–31.9	24.09 ± 3.27 20.06–31.90
IPAQ (METs) Minimum-maximum	44.64 ± 26.18 12.0–92.0	46.43 ± 18.82 17.3–77.8	45.62 ± 21.82 12–92
VO_2*peak*_ (ml⋅kg^–1^⋅min^–1^) Minimum-maximum	50.8 ± 7.5 35.7–59.3	60.0 ± 6.9 51.2–75.7	55.8 ± 8.4 35.7–75.2
HR_max_ (beats⋅min^–1^) Minimum-maximum	184.2 ± 12.2 163.0–201.0	195.6 ± 8.6 180.0–209.0	190.5 ± 11.6 163.0–209.0
**Respiratory values, speed and RPE at OBLA 2.5 mmol/L**			
HR (beats⋅min^–1^) Minimum-maximum	164.2 ± 12.3 143.3–178.2	175.6 ± 8 164.9–188	171.1 ± 11.2 143.3–188.0
% HR_max_	89.0 ± 2.2	89.8 ± 2.5	89.5 ± 2.3
Minimum-maximum	85.8–92.8	85.2–92.8	85.2–92.8
VO_2*peak*_ (ml⋅kg^–1^⋅min^–1^) Minimum-maximum	41.3 ± 5.9 29.7–50.0	45.9 ± 6.0 35.0–52.2	43.8 ± 6.3 29.67–52.2
Speed (km⋅h^–1^) Minimum-maximum	8.5 ± 1.2 6.8–10.1	10.3 ± 1.8 8.0–13.5	9.5 ± 1.8 6.8–13.5
RPE (Arbitrary units) Minimum-maximum	11.7 ± 1.7 8.3–13.8	13.4 ± 1.3 11.0–15.0	12.6 ± 1.7 8.3–15.0

*Data are shown as mean ± SD and range from minimum to maximum.*

*SD, standard deviation; OBLA 2.5 mmol/L, onset blood lactate concentration at 2.5 mmol/L; BMI, Body Mass Index; IPAQ, International Physical Activity Questionnaire; VO_2peak_, Peak Oxygen Uptake; METs, Metabolic Equivalent of Tasks.*

Prior to pre-tests, all participants were informed about the risks and benefits associated with study participation and their right to withdraw from the study at any time without providing any reason, before giving oral and written informed consent. This study was conducted in accordance with ethical standards for health research under the Declaration of Helsinki and the Norwegian Data Protection Service approved the study and storage of personal data (Approval reference number: 584805) without further regional ethical approval per applicable institutional and national guidelines for sport and exercise science.

### Pre-test Procedures

Prior to the pre-tests, the participants answered the International Physical Activity Questionnaire (IPAQ) (Craig et al., 2003) to determine their physical activity level, where we calculated metabolic equivalents of task (MET)-hours per week according to suggested IPAQ scoring methods (Craig et al., 2003). We considered reaching 10.5 MET-hours per week as being physically active (i.e., meeting the minimal global recommendations for physical activity of 150 min per week (Bull et al., 2020). All participants were defined as physically active (lowest = 12 MET-hours per week, highest = 77 MET-hours per week, Table 1). Thereafter, the participants’ body height and mass were measured using a stable stadiometer (Seca 217, Seca GmbH & Co., KG, Hamburg, Germany) and a portable weight scale (Seca 876, Seca GmbH & Co., KG, Hamburg, Germany), respectively, and body mass index (BMI) was calculated (kg/m^2^).

#### Graded Steady State Exercise Test

Following completion of the IPAQ and body mass and height assessment, the participants were fitted with a face mask (COSMED Srl, Rome, Italy) connected to a portable cardiorespiratory analyzer (K5, COSMED Srl, Rome, Italy) attached to the participants’ back and a heart rate (HR) belt (Garmin HRM3, Garmin Ltd., Lathe, KS, United States) was strapped around thorax. The respiratory and HR values were measured each 10 s and transferred via Bluetooth to a portable laptop (ThinkPad, Lenovo Group Ltd, Beijing, China), where we used the manufacturer software (Cardiopulmonary diagnostics Software, COSMED Srl, Rome, Italy) to monitor the measured values. The K5 analyzer was set in mixing chamber mode, which is found to provide valid results for tests to exhaustion when compared to a previously validated stationary cardiorespiratory analyzer (Perez-Suarez et al., 2018). Prior to attaching the K5 to the participants, the analyzer was calibrated with known gas concentrations of oxygen (16%) and carbon dioxide (5%) as well as ambient air (20°C), and the inspiratory flow was manually calibrated against the turbine using a 3 L volume syringe (Calibration Syringe, COSMED Srl, Rome, Italy).

After being equipped with the analyzer and HR belt, the participants entered the treadmill (Woodway ergo ELG 70, Waukesha, WI, United States), which was set to a 5% inclination throughout all tests and exercise sessions. For the first 5 min, the participants warmed up by choosing the speed corresponding to walking or running at a RPE value of 10–14 on Borg’s scale, corresponding to low to moderate exertion (Borg, 1982). The participants thereafter ran for 4 min on the same speed as the last minute of the warmup and subsequently completed a 30 s passive rest to measure BLa from 0.2 μL capillary non-hemolyzed blood sampled from their fingertip on a sterile single-use lancet connected to a mobile lactate analyzer (Lactate Scout +, EKF Diagnostics, Barleben, Germany) and report their RPE. Thereafter, the speed was increased by 1 km⋅h^–1^ and the participants ran for another 4 min before the same BLa measurement routine was carried out. The same procedure was repeated (1 km⋅h^–1^ speed increase, run 4 min, 30 s rest to measure BLa and reported their RPE) until the participants’ BLa reached > 4 mmol/L, in which the test was terminated. BLa above 4 mmol/L was usually reached after 2–4 measurements. The AT was defined as the participants’ running speed associated with a BLa of 2.5 mmol/L (Jamnick et al., 2018), and the exact value was determined by linear interpolation between the measured value below and above 2.5 mmol/L BLa. The same procedure was carried out to determine the HR and RPE corresponding to a 2.5 mmol/L BLa.

#### Test to Exhaustion

Following the graded steady state exercise test, which we considered sufficient as warm up prior to the test to exhaustion, the participants rested for 10 min. Start up speed was set to 1 km⋅h^–1^ under the final speed attained in the incremental steady state test and the participants ran for 1 min where the participants were asked after 45 s whether they could cope with a 1 km⋅h^–1^ increase in 15 s. They indicated a thumb up or down for yes or no, respectively. If they answered yes, the speed was increased with 1 km⋅h^–1^ where the same procedure was repeated (45 s, increase in 15, thumb up or down). If the participants indicated no with a thumb down, they were instructed to keep running on the treadmill until voluntary exhaustion, at which they jumped off the treadmill. Immediately after jumping off the treadmill, the participants reported their RPE. We defined exhaustion as ≥ 17 in RPE and the mean of the three highest consecutive 10 s oxygen uptake (VO_2_) recordings in the test as VO_2*peak*_. All participants reported ≥ 17 in RPE. A test to exhaustion is considered valid for determining HR_max_ (Berglund et al., 2019), where we defined HR_max_ as the highest HR recording in the last minute of the test.

### Exercise Sessions

The week after their pre-tests, the participants reported to the laboratory to start their three exercise sessions per week over 3 weeks (in total nine sessions). Due to logistical reasons, the sessions could be performed within 24 h of a previous session. The exercise sessions were randomized each week, thus controlling for potential confounders, such as exertion from previous exercise. The three treadmill running exercise sessions were (1) CE70: 45 min moderate intensity continuous exercise at 70% of HR_max_, (2) INT80: 4 × 4 min moderate-high intensity interval exercise at 80% of HR_max_ interspaced with 3 min moderate intensity exercise at 70% of HR_max_ between bouts, and (3) INT90: 4 × 4 min high intensity interval exercise at 90% of HR_max_ interspaced with 3 min moderate intensity exercise at 70% of HR_max_ between bouts. The interval sessions were initialized with a 10-min warmup and ended with a 3 min cool down, both at 70% of HR_max_. Consequently, the CE70 lasted in total for 45 min, while the INT80 and INT90 lasted 38 min. The CE70 and INT90 sessions are matched in terms of work (energy expenditure) (Helgerud et al., 2007), and the INT90 and INT80 sessions by time. The participants were unaware of their randomized exercise session until they showed up in the laboratory each day. To monitor exercise intensity, the participants were equipped with a HR belt (Polar H7, Polar Electro Oy, Kempele, Finland) during all sessions, which transferred the HR values to the instructors’ smartphone via Bluetooth and monitored using the manufacturer’s application (Polar Beat, Polar Electro Oy, Kempele, Finland).

We chose to determine the intensity of interval exercise sessions as moderate-high (INT80) and high (INT90) according to HR because this is more accessible to the general population than measuring BLa. The CE70 condition was performed as a reference session, as it is a commonly used exercise session in the literature (Oliveira et al., 2018; Niven et al., 2020; Tavares et al., 2021) and assumed a common exercise modality in the population. In all sessions, the intensity was monitored and controlled (i.e., instructors changed speed if intensity was to high/low according to HR), and the running speed and RPE at 55% completion of each session was registered to calculate relative percentage of AT running speed and AT RPE for each session.

### Outcome Measures

#### Feeling Scale

The participants rated their affective valence (pleasure/displeasure) using the FS before, during (at 55% completion) and immediately after their exercise sessions. The FS is a 11-point scale ranging from –5 (very bad) to 5 (very good) by answering the following question: *“How do you currently feel?”* (Hardy and Rejeski, 1989). Positive and negative affective valence in the FS are found to be associated with a good and bad feeling (Canonical correlation: *r* = 0.87) from the Multiple Affective Adjective Check List, a questionnaire that measures respondents’ feelings (Zuckerman and Lubin, 1985), and shows a moderate negative correlation (*r* = –0.56) with RPE indicating another concept than physical exertion (Hardy and Rejeski, 1989). We translated the FS from English to Norwegian; first, independent translations were performed by two of the authors (TH and ES) and were thereafter compared and no divergent statement was identified. Thereafter, cognitive debriefing of alternative translations were discussed where agreement of final wording was reached and final proofreading was performed (Wild et al., 2005). The translated version can be found in Supplementary Table 2.

#### Physical Activity Enjoyment Scale

The participants filled out the PACES before and after their exercise sessions. The PACES is an 18-item questionnaire, which measures enjoyment of physical activity (Kendzierski and DeCarlo, 1991). The participants rate their agreement with each item ranging from 1 to 7. Negative values (1 as highly agree) were converted to positive values and each item score is summed; the lowest total score is 18 and the highest is 126. The PACES is reported to be valid for internal consistency (Cronbach’s α: 0.96) and reliable (repeatability intraclass correlation coefficient: 0.93) in young adult women and men (Kendzierski and DeCarlo, 1991). The PACES was originally designed to be answered after an activity. We also replaced the original phrase *“Please rate how you feel at the moment about the physical activity you have been doing”* with “… *the activity you are about to start,”* where the participants also answered the PACES prior to their exercise sessions. The PACES is previously translated to Norwegian (Sagelv et al., 2019).

#### Exercise Enjoyment Scale

After 55% completion of the exercise sessions, the participants rated their enjoyment during the exercise using the EES (Stanley and Cumming, 2010). The EES is a 7-point scale ranging from 1 to 7 answering the following statement: *“Use the following scale to indicate how much you are enjoying this exercise session,”* with 1 being *“not at all”* and 7 being *“extremely.”* The EES is reported to be easy to understand and is assumed to be more practical to administrate than longer enjoyment questionnaires over short periods (Stanley and Cumming, 2010), and is reported to be valid (Stanley et al., 2009) when correlated with the interested or enjoyment subscale of the Intrinsic Motivation Inventory (*r* = 0.82–0.85) (Ryan, 1982) and correlates moderately (*r* = 0.41–0.49) with the FS (Hardy and Rejeski, 1989), thus indicating another construct than affect. We translated the EES from English to Norwegian using the same procedure as for translation of the FS (described above) (Wild et al., 2005). The translated version of the EES is found in Supplementary Table 2.

#### Rating of Perceived Exertion

The participants rated their perceived exertion using Borg’s 6–20 RPE scale (Borg, 1974) after 55% of completion and immediately after each exercise session. Borg’s RPE is one of the most used Likert scales for measuring perceived exertion and is consistently found to reflect physiological demands of exercise (Borg, 1974, 1982; Robertson et al., 1998). The RPE ratings were “exercise anchored” (Coquart et al., 2012), where the participants were made familiar with the effort indicating low to moderate intensity from the warmup stage prior to the incremental steady state exercise test, and reported their RPE at each steady state step of the test, and finally reported RPE at maximum intensity following the test to exhaustion.

### Statistical Analyses

We performed 3 by 3 [week (time) × exercise session (CE70, INT80, INT90)] repeated measure univariate analyses of variance (ANOVA) with Bonferonni-corrected *post hoc* tests to assess differences in perceptual responses (FS, EES, PACES, RPE) to the exercise sessions. Effect sizes were calculated as partial eta squared (_*p*_η^2^) where 0.01–0.05, 0.06–0.13, and ≥ 0.14 _*p*_η^2^ were considered small, medium, and large effects, respectively (Richardson, 2011). With three repeated measures (week 1, 2, 3), three exercise sessions (CE70, INT80, INT90), alpha at 0.05 and 80% power, we estimated that 15 participants were needed to observe a large week by exercise session interaction effect (_*p*_η^2^ = 0.14), and 36–18 participants to observe a medium effect (_*p*_η^2^ = 0.06–0.13); 20 participants as in our study needs an effect of _*p*_η^2^ > 0.11. Except for EES “during” and RPE “during” and “after” (all *p* < 0.05), the Shapiro–Wilk test confirmed all outcome variables (FS “before,” “during,” “after,” PACES “before,” “after”) to not deviate from normal distribution (all *p* > 0.10). We considered the repeated measure ANOVAs robust enough to handle the non-normally distributed variables for appropriate interpretations. Sphericity assumptions were confirmed for most analyses (all *p* > 0.05), except for FS “before” and “after” and PACES “before (all *p* ≤ 0.03), where we used Greenhouse–Geisser corrected interpretations (Girden, 1992). We performed sensitivity analyses in the repeated measure ANOVAs by only including those who performed all nine exercise sessions (*n* = 17). We used Pearson correlations to assess the associations between affective valence and enjoyment in exercise sessions and the session’s intensity as individual percentage of AT running speed and AT RPE as all weeks collapsed in total (20 participants × 9 sessions = *n* = 180) and in strata of exercise session (CE70, INT80, INT90; 20 participants × 3 sessions = *n* = 60). We also used Pearson correlations to assess the associations between the mean perceptual responses from all exercise sessions (9 sessions) and BMI, IPAQ scores (MET-hours per week), VO_2*peak*_, individual AT running speed and individual AT RPE (*n* = 20). To reduce false positive rates in the Pearson’s correlations, we adjusted the correlation’s *p*-values according to the Benjamin–Hochberg method (Benjamini and Hochberg, 1995) using 25% false discovery rate. We considered correlations (*r*) of 0.10–0.39, 0.40–69, and ≥0.70 to be small, moderate and large correlations, respectively, which is commonly used effect sizes in psychological research (Akoglu, 2018). Data are shown as mean and 95% confidence intervals (CI) unless otherwise is stated. All statistical analyses were performed using the Statistical Package for Social Sciences (SPSS, version 26, International Business Machine Cooperation, Armonk, NY, United States).

## Results

### Differences in Perceptual Responses to Exercise Sessions

#### Affective Valence

**FIGURE 2:**
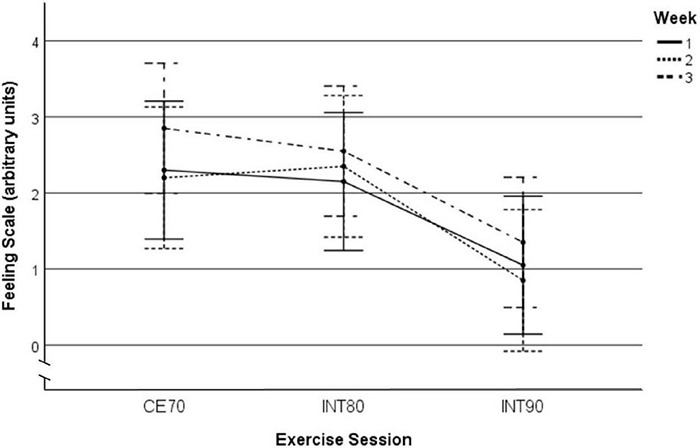
Mean FS score during the exercise sessions in all three weeks separately. Between subject effects. *p* = 0.02, _*p*_η^2^: 0.13. *Post hoc* comparisons: INT90 vs. IN80: *p* = 0.052, INT90 vs. CE70: *p* = 0.03, INT80 vs. CE70: *p* = 1.00. Data are shown as mean and 95% CI. FS, feeling scale; INT90, interval exercise session at > 90% of HR_max_; INT80, interval exercise session at > 80% of HR_max_; CE70, continuous moderate intensity exercise at 70% of HR_max_; CI, confidence interval.

Mean FS scores in all exercise sessions are presented in Table 2. With all sessions collapsed, we observed no changes over the three weeks in FS scores before [main effect of time (weeks); *p* = 0.11, _*p*_η^2^: 0.04], during (*p* = 0.16, _*p*_η^2^: 0.03) or after (*p* = 0.23, _*p*_η^2^: 0.03) the exercise sessions, and there were no differences in changes over weeks by exercise sessions [time (weeks) × exercise session interaction; before: *p* = 0.83, _*p*_η^2^: 0.01; during: *p* = 0.95, _*p*_η^2^: 0.01; after: *p* = 0.93, _*p*_η^2^: 0.01]. We found no differences in mean FS scores of all three weeks (between-sessions’ effects) between exercise sessions before (*p* = 0.77, _*p*_η^2^: 0.02) or after (*p* = 0.23, _*p*_η^2^: 0.03) the exercise sessions, while we observed a difference in mean FS scores during the exercise sessions (*p* = 0.02, _*p*_η^2^: 0.13), where the participants felt lower pleasure during INT90 sessions compared with INT80 (*p* = 0.052) and the CE70 sessions (*p* = 0.03), with no differences between INT80 and CE70 sessions (*p* = 1.00) (Figure 2).

**TABLE 2:** Feeling Scale responses before, during and after the exercise sessions.

		Week 1	Week 2	Week 3	Total#
*FS before*					
CE70	Mean (95% CI)	2.05(1.25−2.85)	1.65(0.73−2.58)	2.15(1.38−2.92)	1.95(1.31−2.59)
INT80	Mean (95% CI)	2.65(1.85−3.45)	2.00(1.08−2.93)	2.10(1.33−2.87)	2.25(1.61−2.89)
INT90	Mean (95% CI)	2.20(1.40−3.00)	1.60(0.68−2.53)	2.18(1.41−2.94)	1.99(1.35−2.63)
*FS during*					
CE70	Mean (95% CI)	2.30(1.39−3.21)	2.20(1.27−3.13)	2.85(2.00−3.71)	2.45(1.72−3.18)
INT80	Mean (95% CI)	2.15(1.24−3.06)	2.35(1.42−3.28)	2.55(1.70−3.41)	2.35(1.62−3.08)
INT90	Mean (95% CI)	1.05(0.14−1.96)	0.85(−0.08−1.78)	1.35(0.50−2.21)	1.08(0.35−1.82)*¤
*FS after*					
CE70	Mean (95% CI)	2.40(1.44−3.36)	2.35(1.40−3.30)	2.70(1.74−3.66)	2.48(1.69−3.28)
INT80	Mean (95% CI)	2.65(1.69−3.61)	2.10(1.15−3.05)	2.55(1.59−3.51)	2.43(1.64−3.23)
INT90	Mean (95% CI)	1.75(0.79−2.71)	1.30(0.35−2.25)	1.78(0.81−2.74)	1.61(0.81−2.41)

*Data are shown as mean and 95% CI. #total as mean of all three weeks.*

*Difference between INT90 versus CE70: p = 0.03.

*¤ Difference between INT90 versus INT80: p = 0.052.*

*FS, feeling scale; CI, confidence interval; CE70, moderate intensity continuous exercise at 70% of HR_max_; INT80, 4 × 4 min intervals at 80% of HR_max_; INT90, 4 × 4 min intervals at 90% of HR_max_.*

#### Enjoyment

**FIGURE 3:**
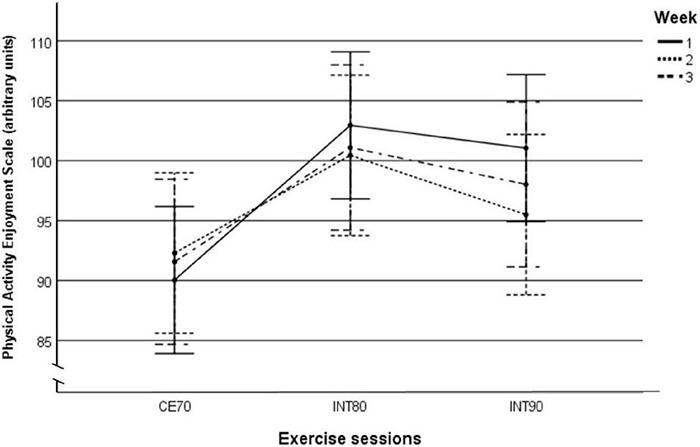
Mean PACES scores after the exercise sessions in all three weeks separately. Between subject effects: *p* = 0.046, _*p*_η^2^: 0.10. *Post hoc* comparisons: INT90 vs. IN80: *p* = 1.00, INT90 vs. CE70: *p* = 0.29, INT80 vs. CE70: *p* = 0.046. Data are shown as mean and 95% CI. PACES, Physical Activity Enjoyment Scale; INT90, interval exercise session at > 90% of HR_max_. INT80, interval exercise session at > 80% of HR_max_; CE70, continuous moderate intensity exercise at 70% of HR_max_; CI, confidence interval.

Mean PACES and EES scores are presented in Table 3. With all sessions collapsed, we observed no changes over the three weeks of PACES scores before [main effect of time (weeks): *p* = 0.36, _*p*_η^2^: 0.02] or after (*p* = 0.47, _*p*_η^2^: 0.01) the exercise sessions, and similarly, we observed no changes over weeks by exercise sessions [time (weeks) × exercise session interaction; before: *p* = 0.64, _*p*_η^2^: 0.02; after: *p* = 0.38, _*p*_η^2^: 0.04]. We found no differences (between-sessions’ effects) in mean PACES scores of all three weeks between exercise sessions before (*p* = 0.42, _*p*_η^2^: 0.03), while we observed a difference in mean PACES scores between exercise sessions after the sessions (*p* = 0.046, _*p*_η^2^: 0.10) where the participants reported higher enjoyment following INT80 sessions compared with CE70 session (*p* = 0.046), but no differences were reported between INT80 and INT90 sessions (*p* = 1.00) or between INT90 and CE70 sessions (*p* = 0.29) (Figure 3).

**TABLE 3:** Physical Activity Enjoyment Scale before- and after- and Exercise Enjoyment Scale during exercise sessions.

		Week 1	Week 2	Week 3	Total#
*PACES Before*					
CE70	Mean (95% CI)	90.70(84.26−97.14)	89.35(82.67−96.03)	89.40(82.68−96.13)	89.82(84.01−95.36)
INT80	Mean (95% CI)	94.75(88.31−101.19)	96.40(89.72−103.08)	93.85(87.13−100.58)	95.00(89.28−100.72)
INT90	Mean (95% CI)	96.45(90.01−102.89)	92.50(85.82−99.18)	91.60(84.88−98.33)	93.52(87.80−99.24)
*PACES After*					
CE70	Mean (95% CI)	90.05(83.92−96.18)	92.30(85.61−98.99)	91.57(84.69−98.46)	91.31(85.54−97.08)
INT80	Mean (95% CI)	102.95(96.82−109.08)	100.45(93.76−107.14)	101.10(94.22−107.99)	101.50(95.73−107.27)*
INT90	Mean (95% CI)	101.05(94.92−107.18)	95.50(88.81−102.19)	98.03(91.14−104.91)	98.19(92.42−103.96)
*EES during*					
CE70	Mean (95% CI)	3.50(3.00−4.00)	4.25(3.60−4.90)	4.20(3.65−4.75)	3.98(3.52−4.45)
INT80	Mean (95% CI)	5.00(4.50−5.50)	4.55(3.90−5.20)	4.75(4.20−5.30)	4.77(4.30−5.23)
INT90	Mean (95% CI)	4.50(4.00−5.00)	4.00(3.35−4.65)	4.33(3.78−4.87)	4.28(3.81−4.74)

*Data are shown as mean and 95%CI.*

*#Total as mean of all three weeks.*

*Difference between INT80 versus CE70: p = 0.46.

*PACES, physical activity enjoyment scale; EES, exercise enjoyment scale; CI, confidence interval; CE70, moderate intensity continuous exercise at 70% of HR_max_; INT80, 4 × 4 min intervals at 80% of HR_max_; INT90, 4 × 4 min intervals at 90% of HR_max_.*

**FIGURE 4:**
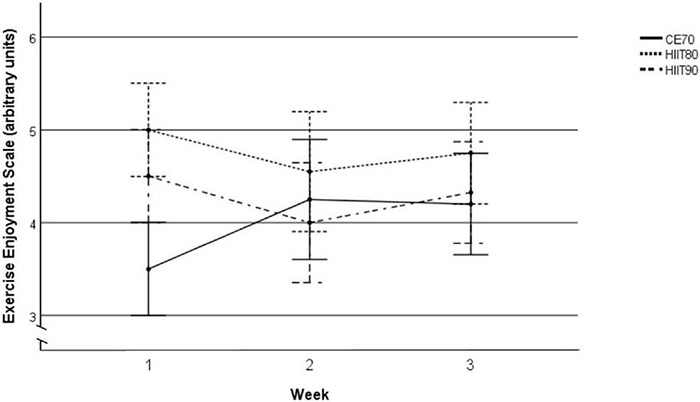
Mean EES score during the exercise sessions in all three weeks separately. Week × exercise session interaction: *p* = 0.01, _*p*_η^2^: 0.11. Data are shown as mean and 95% CI. PACES, Physical Activity Enjoyment Scale; INT90, interval exercise session at > 90% of HR_max_; INT80, interval exercise session at > 80% of HR_max_; CE70, continuous moderate intensity exercise at 70% of HR_max_; CI, confidence interval.

For EES during the exercise sessions, there was no change in enjoyment over the three weeks with all sessions collapsed (main effect of time; *p* = 0.62, _*p*_η^2^: 0.01) but there were differences in changes over weeks by exercise sessions [time (weeks) × exercise session interaction; *p* = 0.01, _*p*_η^2^: 0.11], where participants reported lower enjoyment during the CE70 sessions at week 1 compared with week 2 and 3 (Figure 4). We observed marginally non-significant differences (between-sessions’ effects) in mean EES scores of all three weeks between the exercise sessions (*p* = 0.06, _*p*_η^2^: 0.09), where the participants reported higher enjoyment in INT80 sessions compared with CE70 sessions (*p* = 0.06), with no differences between INT80 vs. INT90 (*p* = 0.42) or between INT90 vs. CE70 (*p* = 1.00).

#### Perceived Exertion

Mean RPE scores for “during” and “after” the exercise sessions are presented in Table 4. With all sessions collapsed, we found no changes in RPE during the exercise sessions over the three weeks (main effect of time; *p* = 0.15, _*p*_η^2^: 0.03), but there was a difference in RPE scores over the three weeks by exercise session [time (weeks) × exercise session interaction; *p* = 0.008, _*p*_η^2^: 0.12], where RPE in INT90 sessions increased from week 1 to week 2, and decreased to week 3 while RPE scores in CE70 and INT80 remained stable. There were differences (between-sessions’ effect) in mean RPE scores of all three weeks between the sessions during exercise (*p* < 0.001, _*p*_η^2^: 0.76), where the participants reported higher exertion by higher intensity sessions (CE70 vs. INT80: *p* < 0.001; CE70 vs. INT90: *p* < 0.001; INT80 vs. INT90: *p* < 0.001). Similarly, for RPE scores after the exercise sessions, there was no changes over weeks with all sessions collapsed (main effect of time; *p* = 0.45, _*p*_η^2^: 0.01) and no changes over weeks by exercise session [time(weeks) × exercise session interaction; *p* = 0.50, _*p*_η^2^: 0.03], but there were differences (between-sessions’ effect) in mean RPE scores of all 3 weeks after the exercise sessions (*p* < 0.001, _*p*_η^2^: 0.71), with higher exertion by higher intensity sessions (CE70 vs. INT80: *p* < 0.001; CE70 vs. INT90: *p* < 0.001; INT80 vs. INT90: *p* < 0.001).

**TABLE 4:** Rating of Perceived Exertion during- and after the exercise sessions.

		Week 1	Week 2	Week 3	Total#
*RPE during*					
CE70	Mean (95% CI)	10.65(10.08−11.22)	10.80(10.19−11.41)	10.53(9.95−11.10)	10.66(10.24−11.08)
INT80	Mean (95% CI)	12.15(11.58−12.72)	11.75(11.14−12.36)	12.15(11.58−12.73)	12.02(11.60−12.44)§
INT90	Mean (95% CI)	13.75(13.18−14.32)	15.10(14.49−15.71)	14.83(14.25−15.40)	14.56(14.14−14.98)*¤
*RPE after*					
CE70	Mean (95% CI)	11.15(10.45−11.85)	10.55(9.76−11.35)	10.30(9.46−11.15)	10.67(10.13−11.21)
INT80	Mean (95% CI)	12.75(12.05−13.45)	12.15(11.36−12.95)	12.55(11.71−13.40)	12.48(11.94−13.02)§
INT90	Mean (95% CI)	15.05(14.35−15.75)	15.30(14.51−16.10)	15.18(14.33−16.02)	15.18(14.64−15.71)*¤

*Data are shown as mean and 95%CI.*

*^#^Total as mean of all three weeks.*

*Difference between INT90 versus CE70: p < 0.001.

*¤ Difference between INT90 versus INT80: p < 0.001.*

*§ Difference between INT80 versus CE70: p < 0.001.*

*RPE, rating of perceived exertion; CI, confidence interval; CE70, moderate intensity continuous exercise at 70% of HR_max_; INT80, 4 × 4 min intervals at 80% of HR_max_; INT90, 4 × 4 min intervals at 90% of HR_max_.*

### Sensitivity Analyses

When including only those who performed all nine exercise sessions (*n* = 17), the results remained generally unchanged (data not shown), except for FS during the exercise sessions, where the differences in mean FS scores of all three weeks between exercise sessions were larger (*p* = 0.001, _*p*_η^2^: 0.17, INT90 versus INT80: *p* = 0.042, INT90 versus CE70: *p* = 0.016, INT80 versus CE70: *p* = 1.00), and for PACES after, where there were no differences in mean PACES scores of all three weeks following sessions (between-sessions’ effect: *p* = 0.09, INT90 versus INT80: *p* = 0.74, INT90 versus CE70: *p* = 0.84, INT80 versus CE70: *p* = 0.09), however, the effect remained unchanged (_*p*_η^2^: 0.10).

### Correlations Between Perceptual Responses and Relative Anaerobic Threshold Running Speed and Relative Anaerobic Threshold Perceived Exertion

**FIGURE 5:**
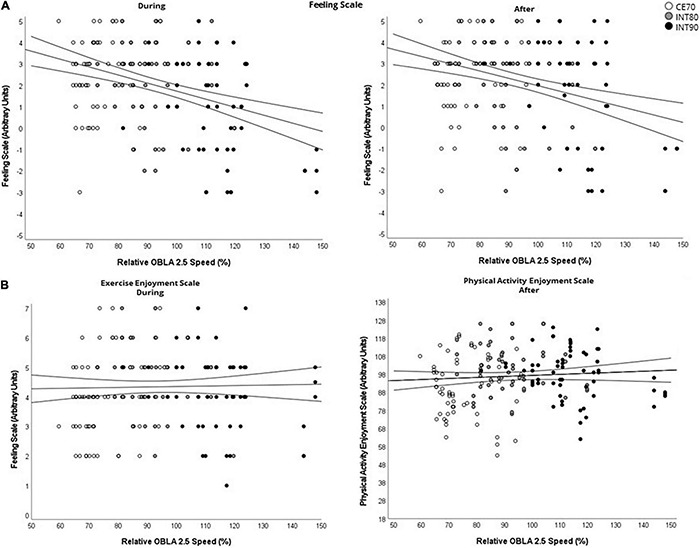
Scatter plots illustrating the relationship between relative OBLA 2.5 running speed across all exercise sessions and weeks (*n* = 180) and **(A)** all FS scores; significant correlation for Feeling Scale during- (Relative OBLA 2.5 Speed: *r* = –0.36, *p* < 0.01) and after the exercise sessions (Relative OBLA 2.5 Speed: *r* = –0.32, *p* < 0.01), **(B)** all PACES and EES scores; no significant correlations. Trend line for regression and 95% CI. OBLA, onset of blood lactate accumulation; CI, confidence interval; FS, feeling scale; PACES, physical activity enjoyment scale; EES, exercise enjoyment scale; RPE, rating of perceived exertion.

Correlations between perceptual responses from all exercise sessions and the participants’ percentage of AT running speed and AT RPE are presented in Table 5, as total (*n* = 180) and in strata of exercise sessions (*n* = 60). Scatter plots of FS during and after, ESS during and PACES after, and the participants’ relative AT running speeds are illustrated in total (*n* = 180) in Figure 5. We found small negative correlations between FS during and after the exercise sessions, and individual relative AT running speed (during: *r* = –0.36, *p* < 0.01; after: *r* = –0.34, *p* < 0.01) (Table 5 and Figure 5). We found moderate positive correlations between RPE during and after the exercise sessions, and individual relative AT running speed (during: *r* = 0.72, *p* < 0.01; after: *r* = 0.72, *p* < 0.01) (Table 5). There were moderate negative correlations between FS and individual relative AT RPE (during: *r* = –0.42, *p* < 0.01; after: *r* = –0.36, *p* < 0.01) (Table 5) and large correlations between RPE and individual AT RPE (during: *r* = 0.70, *p* < 0.01; after: *r* = 0.51, *p* < 0.01) (Table 5).

**TABLE 5:** Correlations of perceptual responses from all exercise sessions and weeks and relative OBLA 2.5 mmol/L speed and RPE.

	Relative OBLA 2.5 mmol/L Speed				Relative OBLA 2.5 mmol/L RPE			
All weeks	All exercise sessions	CE70	INT80	INT90	All exercise sessions	CE70	INT80	INT90
*Number of observations*	*n* = *180*	*n* = *60*	*n* = *60*	*n* = *60*	*n* = *180*	*n* = *60*	*n* = *60*	*n* = *60*
FS During	−0.36**	0.06	−0.18	−0.42**	−0.42**	−0.25	−0.34*	−0.37**
FS After	−0.32**	0.01	−0.25	−0.50**	−0.36**	−0.25	−0.23	−0.44**
PACES After	0.08	−0.04	−0.02	−0.24	−0.10	−0.01	−0.14	−0.57**
EES During	0.02	0.37*	−0.10	−0.26*	−0.08	0.15	−0.14	−0.42**
RPE During	−0.72**	0.19	0.18	0.32*	0.70**	0.60*	0.43**	0.45**
RPE After	−0.72**	0.20	0.37**	0.40*	0.51**	0.23	−0.01	0.20

*Data are correlation coefficients (r) between perceptual responses and relative OBLA 2.5 mmol/L speed and RPE (percentage of individual anaerobic threshold as OBLA at 2.5 mmol/L) and are obtained across all weeks from all exercise sessions as total (20 participants × 9 sessions = 180) and in strata of exercise sessions (CE70, INT80, INT90: 20 participants × 3 sessions = 60).*

*FS, feeling scale; PACES, physical activity enjoyment scale; EES, exercise enjoyment scale; RPE, rating of perceived exertion; OBLA 2.5 mmol/L, onset blood lactate concentration at 2.5 mmol/L blood lactate concentration.*

*Significant correlation at p < 0.05, ^**^ significant correlation at p < 0.01.

For stratified analyses by exercise sessions and the individual percentage of AT running speed, there was a small positive correlation between EES scores during CE70 sessions (*r* = 0.27, *p* = 0.04). In INT80 sessions, we observed a small positive correlation between RPE after the exercise sessions and the participants’ individual AT running speed (*r* = 0.37, *p* < 0.01). In INT90 sessions, we found a moderate negative correlation between FS during (*r* = –0.42, *p* < 0.01) and FS after (*r* = –0.50, *p* < 0.01) the exercise sessions and individual AT running speed. We found a small negative correlation between individual AT running speed and EES during the INT90 sessions (*r* = 0.26, *p* = 0.049) (Table 5).

For stratified analyses by exercise sessions and individual AT RPE, there was a large positive correlation in RPE during CE70 sessions (*r* = 0.60, *p* < 0.01), and a moderate positive correlation in INT80 sessions (*r* = 0.43, *p* < 0.01), and in INT90 sessions (*r* = 0.45, *p* < 0.01). There were moderate negative correlations between relative AT RPE during the exercise sessions and FS after- (*r* = –0.44, *p* < 0.01), EES during (*r* = –0.42, *p* < 0.01), and for PACES after the INT90 sessions (*r* = –0.57, *p* < 0.01) (Table 5).

### Correlations Between Perceptual Responses and Descriptive Participant Data

Correlations between mean perceptual responses from all exercise sessions and weeks, and descriptive participant data (VO_2*peak*_, IPAQ, BMI, AT RPE) are presented in Supplementary Table 1. There were no correlations between perceptual responses and descriptive participant data (all *p* > 0.07), except for a moderate correlation between AT RPE and RPE after (*r* = 0.49, *p* < 0.05).

## Discussion

The main findings in this randomized crossover study were lower affective valence during high intensity interval exercise (INT90) compared with moderate-high intensity interval exercise (INT80) and compared with moderate intensity continuous exercise (CE70). We found higher enjoyment following interval exercise at moderate-high intensity compared with continuous exercise, while no differences were evident between interval exercise at high- and moderate-high intensity. This suggests that structuring the exercise in an interval format could provide a more enjoyable experience and the negative affective consequences associated with high intensity exercise can be alleviated by keeping the intensity below 90% of HR_max_. In addition, we observed negative correlations between affective valance during and after exercise and individual percentage of relative AT running speed. We only observed negative correlations between exercise enjoyment and the individual percentage of relative AT speed in the INT90 sessions.

Our findings corroborate the results of a previous study comparing affect and enjoyment at lower and higher intensities in interval sessions; in 1-min bouts at 70 and 100% of peak work rate (Boyd et al., 2013). Our study expands this work by making such patterns of affect and enjoyment also applicable for long intervals bouts. There are three recent meta-analyses comparing the affective and enjoyment responses from high intensity interval exercise, which consistently report that compared with moderate intensity continuous exercise, higher intensity interval exercise provides lower affective valence (i.e., unpleasantness) during the exercise, while higher enjoyment is reported following the exercise (Oliveira et al., 2018; Niven et al., 2020; Tavares et al., 2021). Our findings corroborate the observations of lower affect during high intensity intervals compared with moderate continuous exercise, but contrary to the consistency in the meta-analyses (Oliveira et al., 2018; Niven et al., 2020; Tavares et al., 2021), we observed no differences in enjoyment following the INT90 and the CE70 sessions. The similar exercise enjoyment observed for INT90 and CE70 is consistent with a previous study comparing similar high intensity long interval bouts of 4 × 4 min with continuous moderate intensity exercise (similar to our CE70) (Sagelv et al., 2019).

However, we observed higher enjoyment following the INT80 sessions compared with the CE70 sessions. Moreover, although non-significant (*p* = 0.06), participants reported higher enjoyment during the INT80 sessions compared with the CE70 sessions, potentially displaying a similar pattern as reported after exercise. This can be attributed to the fact that the exercise modality was intermittent while at the same time performed at moderate-high intensity. Consequently, it is possible to view the interval structure itself as a positive contribution to exercise enjoyment, where our results provide further nuance to this by displaying moderate-high intensity interval exercise as a viable option to produce high enjoyment following exercise, while at the same time produce high pleasure during the exercise.

We observed small negative correlations between relative percentage of individuals’ AT running speed and AT RPE and affect during exercise (Figure 5 and Table 5). These correlations were largely driven by the negative affect reported during the INT90 sessions (Table 5); meaning a higher proportion of the participants’ individual relative AT running speed utilized during exercise had a negative influence on affect (i.e., pleasure) and enjoyment during exercise, which also had negative effect on the affect and enjoyment during the INT90 session with increasing proportion of participants’ relative AT RPE (Table 5). This confirms the notion of lower affective valance as the percentage of individual anaerobic metabolism increases (Ekkekakis et al., 2011; Ekkekakis and Brand, 2019), which also applies for interval exercise.

We observed a week by exercise modality interaction for exercise enjoyment during exercise, where enjoyment in moderate intensity continuous exercise increased while enjoyment in the interval exercise sessions remained stable over 3 weeks (Table 3). The stable enjoyment in interval exercise is in line with two previous studies that examined enjoyment responses over 3 (Vella et al., 2017) and 5 weeks (Kong et al., 2016). In contrast, another study found enjoyment to progressively increase over the study duration, where higher enjoyment was reported in high intensity intervals compared with moderate intensity continuous exercise from week 4 to 6 (Heisz et al., 2016). Due to the COVID-19 lockdown, we were forced to cut our intervention short by 1 week, thus we were unable to assess whether our exercise sessions would differ by week 4. However, considering that we observed no effect of time to indicate any progressively increased enjoyment within week 3, a difference in week 4 seems unlikely. Nevertheless, our study differs from the previous studies (Heisz et al., 2016; Kong et al., 2016; Vella et al., 2017) by design; their designs were randomized paralleled groups while our study was a randomized crossover trial. Crossover designs use the same individuals as controls, thus removing a potential influence of individual variation between groups (Sibbald and Roberts, 1998). It also results in multiple treatment effects, where participants in our study had past references (experience) from all exercise session following week 1, which may have resulted in higher enjoyment during CE70 sessions in week 2 and 3 (Figure 4). Thus, with previous experience from high intensity intervals, moderate intensity exercise might be perceived more enjoyable as a result of lower positive affect from past experience with high intensity intervals (Ekkekakis et al., 2011; Ekkekakis and Brand, 2019).

Interestingly, there seem to be strong debates on whether high intensity intervals are appropriate for the vast majority of the population (Biddle and Batterham, 2015). A previous study indicated higher adherence in moderate intensity continuous exercise (similar to our CE70, 75% adherence) compared with 4 × 4 min intervals (60% adherence) following 12 weeks (Lunt et al., 2014). In a recent larger study (*N* = ∼800), exercise adherence seems similar between 4 × 4 min intervals and moderate continuous exercise following both 1 year (∼50-60% adherence in both groups), 3 and 5 years (∼50% adherence) (Stensvold et al., 2020), indicating that both exercise modalities are equally difficult to adhere to. Consequently, it may be that exercise *per se* is difficult to sustain over time, independent of exercise mode or modality.

Difference in exercise adherence between high intensity intervals and moderate intensity continuous exercise may be due to inappropriate delivery of high intensity interval exercise. In the study by Lunt et al. (2014), they instructed their participants to reach target HR within 10–20 s into the interval bouts. Due to heart rate kinetics at the onset of exercise, this likely involved high anaerobic metabolism and thus large accumulated BLa throughout the entire exercise session, with recovery periods between bouts too short for accumulated BLa clearance (Jones et al., 2010). Appropriately delivered high intensity long interval bouts allows the first interval bout to reach target HR at the end of the 4 min, thereafter, following 2 min in the remaining interval bouts (Karlsen et al., 2017; Taylor et al., 2019), as done by Stensvold et al. (2020). As such long interval bouts are designed not to be totally exhaustive, individual changes should be made if RPE ratings reaches > 17, which indicates too exhaustive interval bouts (Taylor et al., 2019). For example, the 4 min intervals of 80 and 90% of HR_max_ in our study was rated as 13 and 15 in RPE, respectively (Table 4), and in the study by Stensvold et al. (2020) as 13 (CE70) and 16 (INT90), respectively, corresponding to “somewhat hard” to “hard” exertion (Borg, 1974). Thus, the lower exercise adherence of high intensity as observed in the study by Lunt et al. (2014) may be due to inappropriately delivered high intensity long interval bouts, not the high intensity long interval bouts *per se*. Nevertheless, even if cardiac drift is taken into account, it still seems that short interval bouts (60 s) at HR > 90% of HR_max_ results in lower adherence compared with intervals at ≤ 85% of HR_max_ (Arad et al., 2020), which is supported by our study where higher positive affect was observed in long interval bouts aimed at eliciting 80% of HR_max_.

We observed no association between descriptive participant data (cardiorespiratory fitness, body mass index and physical activity level) and perceptual responses of exercise, which is in line with previous literature (Oliveira et al., 2018). As affective valance is associated with physical activity (Williams et al., 2012), we included physical activity level as descriptive participant data, which most previous studies did not measure, but mentioned as “inactive” or “active” *etc.* (Oliveira et al., 2018; Niven et al., 2020; Tavares et al., 2021). Although we observed no association between physical activity level and perceptual responses of exercise, the participants in our study reported to be highly active, where some individuals were determined to be up to four times more active than the minimal recommendations for physical activity of 150 min (Bull et al., 2020), which is far beyond the general population (Guthold et al., 2018). Thus, although we observed no differences between perceptual responses and physical activity levels, there may be actual differences in perceptual scores (potentially between inactive and active) that our study is unable to disentangle, as observed previously for body mass index between obese and non-obese women (Ekkekakis et al., 2010). Nevertheless, it is still intriguing to observe that the perceptual responses, especially during exercise, are similar to what has been reported previously for both active and inactive individuals (Oliveira et al., 2018; Niven et al., 2020; Tavares et al., 2021), suggesting that the pattern of perceptual responses in relation to relative exercise intensity are similar across physical activity levels. Consequently, our findings of the pattern for perceptual responses toward exercise are likely representable for those with lower physical activity level.

Due to low cardiorespiratory fitness in many populations (Aspenes et al., 2011; Edvardsen et al., 2013; Kaminsky et al., 2015), some have suggested that exercise professionals face challenges in finding appropriate activities for individuals with low cardiorespiratory fitness, thus being applicable for the unsustainability of higher intensity intervals (Decker and Ekkekakis, 2017). Although this is true, it is far from impossible; this depends on appropriately educated exercise professionals using relative intensity measures alongside flexible approaches to adapt the exercise according to perceptual responses (Taylor et al., 2019). Consequently, those with low cardiorespiratory fitness may simply walk at an absolute intensity corresponding to e.g., 1 km⋅h^–1^, which can correspond to 10–90% of HR_max_ depending on their cardiorespiratory fitness level (Biddle and Batterham, 2015).

Finally, no associations between descriptive participant data and affective- and enjoyment responses indicate that other factors cause the observed pattern in these responses to high intensity exercise. Physiological reasons are likely related to anaerobic metabolism (Ekkekakis and Brand, 2019). Psychological suggestions for the heterogeneous responses may include self-efficacy, or social evaluations such as appearance/social physique anxiety (Ekkekakis and Brand, 2019). Moreover, perceptual responses are likely influenced by social and cultural contexts, which involve interwoven cognitive evaluations and feelings that can be both competence- and body-related (Ekkekakis and Brand, 2019). For example, intrinsic motivation for exercise is found to mediate the association between affect and exercise (Schneider, 2018) and a previous twin study indicate that affective responses may be, to some extent, heritable (Schutte et al., 2017). Such psychological factors warrant further investigation into mechanisms that can predict exercise adherence.

### Strengths

To our knowledge, this is the first study to compare affective valence and exercise enjoyment of long interval bouts at different intensities. By comparing these two (INT80 and INT90), and the traditional moderate intensity continuous exercise (CE70), it is possible to elucidate the effect of structuring the exercise in long interval bout formats allowing for higher stroke volume adaptations (Bacon et al., 2013) with lower interference of the anaerobic processes associated with high intensity. Furthermore, as mentioned above, our randomized crossover design over three weeks allowed the participants to rate their affect and enjoyment with previous experience from all three exercise sessions, while also controlling for interindividual differences. Finally, due to the many exercise sessions, we were able to display associations between individual percentage of the AT (running speed and RPE at OBLA 2.5 mmol/L) and perceptual responses during and after exercise, which corroborate the observation observed previously in continuous exercise, where participants experience lower affect as the intensity exceeds physiological landmarks of relative higher contribution from anaerobic metabolism (Ekkekakis et al., 2011; Ekkekakis and Brand, 2019).

### Limitations

Compared with previous studies in recent meta-analyses (Oliveira et al., 2018; Niven et al., 2020), our participants were relatively fit in terms of cardiorespiratory fitness, body mass index and physical activity level. However, there seem to be no association between fitness level and perceptual reporting during and after exercise (Oliveira et al., 2018). As mentioned above, our participants were highly active, where differences in actual responses (e.g., affective and enjoyment scores) may differ although likely produce similar patterns in aerobic exercise modalities. Nevertheless, our study should be replicated in individuals of lower physical activity levels to draw firmer conclusions on patterns and responses toward different exercise intensities and modalities in that population.

Furthermore, we measured affect once during the exercise sessions, at 55% completion. This puts our affect assessment between interval bout two and three in the interval sessions. Some have suggested that the FS should be measured multiple times capturing both peak intensity and peak recovery time at all interval bouts (Decker and Ekkekakis, 2017), and this may be regarded as a limitation to our study. However, as perceived exertion is influenced by both intensity and duration of exercise (Garcin and Billat, 2001), recovery periods between interval bouts will still capture exertion, thus also likely unpleasantness, within the exercise. Thus, measuring affect immediately following the second interval bout allowed us to capture the associated affect during the exercise, where we observed similar patterns as anticipated of the long interval bouts at different intensities. Similarly, presenting the EES and the FS at the same time during an exercise is suggested to influence the FS scores as the EES is a positive loaded question (“*how much are you enjoying*…”) (Ekkekakis et al., 2011; Decker and Ekkekakis, 2017); this may also be regarded as a limitation. To address this, we first presented the FS followed by the EES to the participants.

We included 20 participants, which are borderline sufficient statistical power to observe a “high” medium (_*p*_η^2^ ≥ 0.11) time × exercise condition interaction, but sufficient to observe a large effect (_*p*_η^2^ ≥ 0.14). There might be smaller effects that were not detectable in our study and cannot be ruled out based on this investigation. For example, the marginally non-significant (*p* = 0.06) difference in enjoyment during INT80 compared with CE70 can be assessed with higher certainty in a larger sample. However, a previous study reported a 1-unit increase in the FS during exercise to translate to 15 min higher physical activity at 6 months follow-up (Williams et al., 2012); as our FS differences during exercise are > 1-unit between exercise sessions (Table 2 and Figure 2), it can be considered a relevant difference.

As we produced 180 observations for our correlation analyses between individual percentage of AT speed and AT RPE from nine exercise sessions in 20 participants, this likely caused autocorrelation (i.e., similar correlations within each individual but at different time points; week 1 and session 1, week 2 and session 4 etc.). However, as our repeated randomized crossover design allowed for past experience from all exercise sessions, this may have changed the associations over time, thus in our view justifying these analyses. Moreover, we chose OBLA at 2.5 mmol/L as our AT. There are great controversies and confusion on whether one can pinpoint an AT, or whether OBLA-determined thresholds are reproducible (Jamnick et al., 2018; Poole et al., 2021). However, we chose a feasible estimate of an anaerobic transition, allowing us to display the associations between perceptual responses during and after exercise with individual percentage of AT intensity, where our results were in line with previous literature using continuous exercise (Ekkekakis et al., 2011; Ekkekakis and Brand, 2019).

Furthermore, three participants were unable to complete their final exercise session (two INT90 and one CE70) due to the COVID-19 lockdown, where we forwarded their respective mean score (CE70 or INT90) of week 1 and 2 to their respective missing exercise session in week 3. Thus, possible changes in perceptual responses by week three for these individuals could potentially influence our results. However, as our sensitivity analyses were generally unchanged when including only the 17 participants that completed all nine exercise sessions, it is unlikely that this influenced our interpretation of the results.

Finally, although our intervention were three weeks long, such a time span is not long enough to assess any meaningful exercise adherence. Well-designed long-term studies (i.e., 3–6–12 months) assessing perceptual responses and exercise adherence with different exercise mode and/or modalities are warranted.

## Conclusion

Structuring the exercise in an interval format could provide a more enjoyable experience and the negative affective consequences associated with high intensity exercise can be alleviated by keeping the intensity at or around 80% of HR_max_, which may contribute to higher exercise adherence and consistency.

## Data Availability Statement

The original contributions presented in the study are included in the article/Supplementary Material, further inquiries can be directed to the corresponding author/s.

## Ethics Statement

Ethical review and approval was not required for the study on human participants in accordance with the local legislation and institutional requirements. This study does not fall under the Health Research Act in Norway (https://lovdata.no/dokument/NL/lov/2008-06-20-44). The patients/participants provided their written informed consent to participate in this study.

## Author Contributions

TH, ES, and SP designed the study. TH and ES translated the FS and EES questionnaires. ES performed all statistical analyses and wrote the methods and results. TH was in charge of the writing process and wrote the introduction and discussion. All authors revised, read and approved the final version of the manuscript.

## Conflict of Interest

The authors declare that the research was conducted in the absence of any commercial or financial relationships that could be construed as a potential conflict of interest.

## Publisher’s Note

All claims expressed in this article are solely those of the authors and do not necessarily represent those of their affiliated organizations, or those of the publisher, the editors and the reviewers. Any product that may be evaluated in this article, or claim that may be made by its manufacturer, is not guaranteed or endorsed by the publisher.

## Supplementary Material

Supplementary Table 1 Correlation matrix of mean perceptual responses from all exercise sessions and weeks and descriptive participant data, as total.

Supplementary Table 2 Translated version of Feeling Scale (FS) and translated version of Exercise Enjoyment Scale (EES).

Supplementary File 1 The dataset for this study.

## Abbreviations

INT90: 4 × 4 min intervals at 90% of heart rate maximum
INT80: 4 × 4 min intervals at 80% of heart rate maximum
CE70: moderate continuous exercise at 70% of heart rate maximum
AT: anaerobic threshold
OBLA: onset of blood lactate accumulation
PACES: physical activity enjoyment scale
EES: exercise enjoyment scale
FS: feeling scale
RPE: rating of perceived exertion.
