# Supplementary material for: Affective Valence and Enjoyment in High- and Moderate-High Intensity Interval Exercise. The Tromsø Exercise Enjoyment Study
Source: Front Psychol. 2022 Mar 22;13:825738. doi: 10.3389/fpsyg.2022.825738 (PMC8982257; doi:10.3389/fpsyg.2022.825738)
Supplement: Supplementary Table 1 — Correlation matrix of mean perceptual responses from all exercise sessions and weeks and descriptive participant data, as total. [file Table_1.docx]

**Supplementary Table 1.** Correlations between mean perceptual responses of all exercise sessions and weeks and descriptive participant data.

| **N=20** | **VO_2peak_** | **IPAQ** | **BMI** | **OBLA 2.5 mmol∙L^-1^ Speed** | **OBLA 2.5 mmol∙L^-1^ RPE** | **OBLA 2.5 mmol∙L^-1^ VO_2_** |
| --- | --- | --- | --- | --- | --- | --- |
| *All sessions* |  |  |  |  |  |  |
| FS Before | 0.06 | -0.21 | -0.15 | 0.08 | -0.05 | 0.33 |
| FS During | 0.04 | -0.32 | 0.04 | 0.01 | 0.20 | 0.36 |
| FS After | -0.01 | -0.29 | 0.17 | -0.04 | 0.27 | 0.32 |
| PACES Before | 0.21 | 0.12 | 0.15 | 0.22 | 0.25 | 0.21 |
| PACES After | 0.10 | -0.09 | 0.14 | 0.08 | 0.20 | 0.13 |
| EES During | -0.09 | -0.41 | -0.05 | -0.16 | 0.01 | 0.16 |
| RPE During | -0.06 | -0.21 | 0.31 | -0.18 | 0.41 | -0.28 |
| RPE After | 0.14 | -0.09 | 0.13 | 0.06 | 0.49* | -0.13 |

Data are correlation coefficients (*r*) between the mean from all exercise sessions from all weeks. FS=Feeling Scale, PACES=Physical Activity Enjoyment Scale, EES=Exercise Enjoyment Scale, RPE=Rating of Perceived Exertion, BMI=Body Mass Index, IPAQ=International Physical Activity Questionnaire, VO_2peak_=Peak Oxygen Uptake, VO_2_=Oxygen Uptake, OBLA 2.5 mmol/L Speed=Speed at 2.5 mmol/L onset blood lactate accumulation, OBLA 2.5 mmol/L RPE=RPE at 2.5 mmol/L onset blood lactate accumulation, OBLA 2.5 mmol/L VO_2_=oxygen uptake at 2.5 mmol/L onset blood lactate accumulation. *Significant correlation at p<0.05.
