# Supplementary material for: Affective Valence and Enjoyment in High- and Moderate-High Intensity Interval Exercise. The Tromsø Exercise Enjoyment Study
Source: Front Psychol. 2022 Mar 22;13:825738. doi: 10.3389/fpsyg.2022.825738 (PMC8982257; doi:10.3389/fpsyg.2022.825738)
Supplement: Supplementary Table 2 — Translated version of Feeling Scale (FS) and translated version of Exercise Enjoyment Scale (EES). [file Table_2.docx]

**Feeling scale:**

Hardy, C.J., and Rejeski, W.J. (1989). Not What, but How One Feels: The Measurement of Affect during Exercise. 11(3)**,** 304. doi: 10.1123/jsep.11.3.304.

*“Under deltagelse i denne treningen kan du oppleve ulike variasjoner i humør. For eksempel vil enkelte oppleve treningen som gledelig, mens andre opplever den som ubehagelig.*

*På nåværende tidspunkt, i hvilken grad føler du det behagelig eller ubehagelig?»*

| -5 | -4 | -3 | -2 | -1 | 0 | 1 | 2 | 3 | 4 | 5 |
| --- | --- | --- | --- | --- | --- | --- | --- | --- | --- | --- |
| Veldig dårlig |  | Dårlig |  | Ganske/litt dårlig | Nøytral | Ganske/litt bra |  | Bra |  | Veldig bra |

**Exercise Enjoyment scale**

Stanley DM, Cumming J. Are we having fun yet? Testing the effects of imagery use on the affective and enjoyment responses to acute moderate exercise. Psychology of Sport and Exercise. 2010;11(6):582-90.

*“Bruk følgende skala til å indikere hvor mye du nyter denne treningsøkten.»*

| 1 | 2 | 3 | 4 | 5 | 6 | | 7 |
| --- | --- | --- | --- | --- | --- | --- | --- |
| Veldig lite | Litt | Moderat | | Ganske mye | | Veldig mye | |

**Physical Activity Enjoyment Scale 18-items, 7-point bipolar scale**

Kendzierski, D., and DeCarlo, K.J. (1991). Physical Activity Enjoyment Scale: Two Validation Studies. *Journal of Sport & Exercise Psychology* 13(1)**,** 50. doi: 10.1123/jsep.13.1.50 10.1123/jsep.13.1.50.

*Translated to Norwegian in:*

Sagelv, E.H., Hammer, T., Hamsund, T., Rognmo, K., Pettersen, S.A., and Pedersen, S. (2019). High Intensity Long Interval Sets Provides Similar Enjoyment as Continuous Moderate Intensity Exercise. The Tromsø Exercise Enjoyment Study. *Frontiers in Psychology* 10(1788). doi: 10.3389/fpsyg.2019.0178

*indikerer motsatt score: 1 = 7, 2 = 6, 3 = 5, 4 = 4, 5 = 3, 6 = 2, 7 = 1.

| *Jeg nyter det | 1 | 2 | 3 | 4 | 5 | 6 | 7 | Jeg hater det |
| --- | --- | --- | --- | --- | --- | --- | --- | --- |
| Jeg føler kjedsomhet |  |  |  |  |  |  |  | Jeg føler meg interessert |
| Jeg missliker det |  |  |  |  |  |  |  | Jeg liker det |
| Jeg synes det er behagelig |  |  |  |  |  |  |  | Jeg synes ikke det er behagelig |
| Denne aktiviteten tiltrekker meg |  |  |  |  |  |  |  | Denne aktiviteten er ikke i det hele tatt tiltrekkende for meg |
| Det er ikke morsomt i det hele tatt |  |  |  |  |  |  |  | Det er veldig moro |
| Jeg synes det er energigivende |  |  |  |  |  |  |  | Jeg synes det er utmattende |
| Det gjør meg depressiv |  |  |  |  |  |  |  | Det gjør meg glad |
| *det er veldig hyggelig |  |  |  |  |  |  |  | Det er veldig uhyggelig |
| *Jeg føler meg bra fysisk når jeg gjør det |  |  |  |  |  |  |  | Jeg føler meg dårlig fysisk når jeg gjør det |
| *Det er veldig styrkende |  |  |  |  |  |  |  | Det er ikke styrkende i det hele tatt |
| jeg er veldig frustrert av det |  |  |  |  |  |  |  | Jeg er ikke frustrert av det i det hele tatt |
| *det er veldig gledelig |  |  |  |  |  |  |  | Det er ikke gledelig i det hele tatt |
| *det er veldig spennende |  |  |  |  |  |  |  | Det er ikke spennende i det hele tatt |
| Det er ikke stimulerende i det hele tatt |  |  |  |  |  |  |  | Det er veldig stimulerende |
| *det gir meg en sterk følelse av gjennomføring |  |  |  |  |  |  |  | Det gir meg ikke en sterk følelse av gjennomføring |
| *det er veldig forfriskende |  |  |  |  |  |  |  | Det er ikke forfriskende i det hele tatt |
| Jeg følte det slik at jeg heller ville gjøre noe annet |  |  |  |  |  |  |  | Jeg følte det slik at det er ingen ting annet jeg heller ville gjort |
